# Supplementary material for: Physical properties of lactic acid bacteria influence the level of protection against influenza infection in mice
Source: PLoS One. 2021 May 18;16(5):e0251784. doi: 10.1371/journal.pone.0251784 (PMC8130949; doi:10.1371/journal.pone.0251784)
Supplement: Attachment — Submitted filename: Comments.docx [file pone.0251784.s001.docx]

In this study, the authors investigated how the powderization of lactic acid bacteria (LAB) affects their water dispersibility and their efficacy in inducing immune response in a mouse model of IFV infection. Two different types of powdered *Enterococcus faecalis* KH2 preparation (non-treated and dispersed) were compared in their particle size, the distribution in the small intestine and the protective effects of these LAB preparations against viral infection in a mouse model of influenza infection. The authors showed that by treating LAB with a high-pressure homogenizer and adding dextrin in the preparation can dramatically decrease the formation of bacteria aggregates, which increased the water dispersibility of the bacteria and thus enhanced their uptake by intestinal Peyer’s patches and improved their protection against virus infection. The experiments were well-designed, and the conclusion was reasonably sound. However, there are several major concerns that should be addressed:

1. Several abbreviations were not defined in the manuscript and should be added in the main text where it first appeared (eg. PRRs, M cells, et al).
2. Line 62, “to investigate whether the effect of the difference in the LAB species or powderization was due to the difference in the species” This statement is really confusing and should be rephrased. Also, were the species of bacteria in the LAB powdered preparation different in n-LAB and d-LAB?
3. Typos and grammar issues should be double-checked and corrected.
4. In the evaluation of LAB’s distribution in mouse Peyer’s patches, male specific pathogen-free mice were used, whereas female mice were used in the influenza infection model. Are there any particular reasons in choosing female mice in the infection model? And have you looked at the bacteria distribution in Peyer’s patches in female mice as well? Is it different from male mice, can you comment on this?
5. The distribution of LAB in mouse intestine was evaluated in male mice injected with LAB and harvested after 1h in this manuscript, LAB preparations were also administered to female mice by gavaging in the IFV infection model. Have you looked at the distribution of LAB in mice with IFV infection? How does the distribution change overtime before and after virus infection? Can you comment on this?
6. Line 99, the LAB suspension was added “to six wells of a 96-well cell culture plate”, is this correct?
7. In the IFV infection model, n-LAB, d-LAB and OSL were given two times per day from 7 days before viral inoculation to 14 days after inoculation. Were there any differences in body weight change in the first 7 days with LAB administration before viral inoculation? Have you tried different treatment schemes (pre-treatment, LAB administration during infection, or LAB after infection) with LAB preparations in the infection model and were there any differences?
8. Figure legends should be placed following the figure or combined and placed after the main text of the manuscript.
9. Virus loads in BALFs and lungs were determined on day 3 after IFV infection and neutralizing antibody and IgA levels were determined on day 14. Have you tested the virus load in BALFs and lungs on day 3 as well?
10. In Figure 3, a and b seems showing same magnification based on the scale bar, however the images seem to be taken under obviously different magnification scale, this should be addressed and corrected, if any applicable. Additionally, the indications of different fluorescence colors presenting in Figure 3c should be added in the figure legend.
11. Line 181-184. This part of result was written poorly. The rationale and hypothesis of this specific experiments set should be addressed and a conclusion from the observations should be made or at least discussed.
12. Influenza infected mice treated with d-LAB lost weight at a slower rate and recovered faster compared to those treated with n-LAB, was the difference statistically significant? The signifiers should be added to the graphs if there is any.
13. Figure 6a, significance bar misplaced. Please correct this.
14. Line 207, The d-LAB group had a significantly lower viral load than n-LAB group in the BALFs but no differences were seen in the lung virus yields, can you explain this?
15. A control group of influenza infected mice treated with LAB culture preparation without powderization should be added, in comparison to different types of powdered LAB preparations.
16. Are there any other aspects other than the formation of aggregates that could possibly affect the beneficial effects of LAB? (eg, bacteria morphology changes during and after powderization, biofilm formation, et al.)
