# Supplementary material for: Physical properties of lactic acid bacteria influence the level of protection against influenza infection in mice
Source: PLoS One. 2021 May 18;16(5):e0251784. doi: 10.1371/journal.pone.0251784 (PMC8130949; doi:10.1371/journal.pone.0251784)
Supplement: Attachment — Submitted filename: Response to reviewers.docx [file pone.0251784.s002.docx]

Dr. Wenke Feng

Academic Editor

*PLOS ONE*

January 25, 2021

Manuscript reference ID: PONE-D-20-33627

Dear Editor:

Please find attached a revised version of our manuscript “Physical properties of lactic acid bacteria influence the level of protection against influenza infection in mice,” which we would like to resubmit for publication as a *Research Article* in *PLOS ONE*.

The comments provided by you and the reviewers were highly insightful and enabled us to greatly improve the quality of our manuscript. Our point-by-point responses to each of the comments raised are provided below.

Revisions in the manuscript are shown as blue font for additions and revisions and as stricken through text (~~example)~~ for deletions. We hope that the revisions in the manuscript and our accompanying responses would sufficiently address your concerns and make our manuscript suitable for publication in *PLOS ONE*.

We look forward to hearing from you at your earliest convenience.

Yours sincerely,

Takumi Watanabe

Address: Chubu University, Graduate School of Engineering, Kasugai, Aichi, JP

E-mail: [t.watanabe@bio-ken.jp](mailto:t.watanabe@bio-ken.jp)

**Responses to Reviewer #1:**

We thank the Reviewer for the insightful comments. We believe that the comments have helped us significantly improve the paper. In particular, we would like to express our gratitude for the opportunity to consider the mechanism of action of lactic acid bacteria in further detail.

1. The microscopy seems to be of poor quality and it is hard to assess in the images the location of the intestinal cells in the background. The confocal microscopy is somewhat better but it is necessary to show the results of the aggregated bacteria on confocal microscopy as well as the dispersed.

Response: An image from the lumen side was added to illustrate the relationship between intestinal cells and bacteria (Fig 3c). Because n-LAB (aggregated bacteria) cannot be observed on epithelial cells of Peyer’s patches, we did not detect them using confocal microscopy. As mentioned by you, a comparison between aggregated and dispersed bacteria should be performed; accordingly, we intend to collect the Peyer’s patches and count the number of bacteria in future research.

2. It should be elucidated what the basis for assuming that the bacterial mechanism of action mainly involved binding to Peyer's Patches.

Response: Thank you for your important comment. We have added that bacterial S-layer protein is absorbed via binding to uromodulin of M cells in Peyer’s patches [L57-59]. A reference of previous studies [14] has been added.

3. It should be made clear in the discussion that the theory that the aggregated bacteria cannot traverse mucin layers in speculative.

Response: As mentioned by you, it is a speculation; accordingly, we intend to conduct research on the mucus on the Peyer's patches to determine if there is a difference in passage of particles owing to aggregation. We have added content to clarify this [L283-284].

4. The discussion also notes that there is different interaction of the dispersed bacteria with macrophages and dendritic cells but it is not clear from the data presented that these are the cells involved in generating the IL-12.

Response: The description of the cells used (mouse splenocytes) was incorrect and has been rectified [L286-287].

5. There should be some discussion of why the splenocyte and IL-12 experiments were done.

Response: Thank you for this comment. To clarify this, in the Introduction, we have added a sentence about verifying the effect of bacterial aggregation on IL-12 production using mouse splenocytes and two appropriate references [L77-78].

6. It should be made clear if the rate of recovery of weight was statistically different for the dispersed bacteria.

Response: There was no significant difference in weight recovery of mice in the d-LAB group when compared with other groups or during the 7-day administration period. We have added sentences to clarify this [L221-225].

7. Figure 4 title refers to ribonuclease treatment - Where does the ribonuclease come in?

Response: We apologize for the error and have rectified it [L209].

**Responses to Reviewer #2:**

We wish to express our appreciation to the Reviewer for the insightful comments, which have helped us significantly improve the paper. We also thank you for your valuable suggestions for future research.

1. Several abbreviations were not defined in the manuscript and should be added in the main text where it first appeared (eg. PRRs, M cells, et al).

Response: Thanks for bringing this to our notice. We have rectified these instances (PRRs, M cell, and DAPI).

2. Line 62, “to investigate whether the effect of the difference in the LAB species or powderization was due to the difference in the species” This statement is really confusing and should be rephrased. Also, were the species of bacteria in the LAB powdered preparation different in n-LAB and d-LAB?

Response: We agree that the context was difficult to understand and apologize for the same. We have revised it [L66-71]. n-LAB and d-LAB are the same strain of bacteria.

3. Typos and grammar issues should be double-checked and corrected.

Response: We asked a private proofreading company (Enago) to re-check the manuscript and rectify such errors.

4. In the evaluation of LAB’s distribution in mouse Peyer’s patches, male specific pathogen-free mice were used, whereas female mice were used in the influenza infection model. Are there any particular reasons in choosing female mice in the infection model? And have you looked at the bacteria distribution in Peyer’s patches in female mice as well? Is it different from male mice, can you comment on this?

Response: Female mice were used in these experiments because they have been used in our past influenza infection studies and other studies. One of the reasons for this is that female mice have a calmer temperament, which reduces the stress on the mice and the experimenter during continuous administration. We have not assessed bacterial distribution in female mice in the present study; however, we have previously confirmed that they were absorbed by the Peyer’s patches in the other experiments using the same species of BALB/c mice. However, as mentioned by you, there may be differences between males and females; accordingly, we intend to clarify the differences between males and females in future studies.

5. The distribution of LAB in mouse intestine was evaluated in male mice injected with LAB and harvested after 1h in this manuscript, LAB preparations were also administered to female mice by gavaging in the IFV infection model. Have you looked at the distribution of LAB in mice with IFV infection? How does the distribution change overtime before and after virus infection? Can you comment on this?

Response: We have not observed any distribution of LAB in IFV-infected mice. As mentioned in your comment, IFV infection may have changed the distribution; thus, we would like to confirm the effect of infection and distribution in future studies.

6. Line 99, the LAB suspension was added “to six wells of a 96-well cell culture plate”, is this correct?

Response: We apologize for the confusing notation. It has been corrected to “6 wells per sample” [L109].

7. In the IFV infection model, n-LAB, d-LAB and OSL were given two times per day from 7 days before viral inoculation to 14 days after inoculation. Were there any differences in body weight change in the first 7 days with LAB administration before viral inoculation? Have you tried different treatment schemes (pre-treatment, LAB administration during infection, or LAB after infection) with LAB preparations in the infection model and were there any differences?

Response: There were no differences in body weight change in each group during the 7 days of administration pre-infection. This information has been added to the text [L224-225]. The comparison of lactic acid bacteria in different treatment schemes is of interest to us and we will continue our research to further clarify these points.

8. Figure legends should be placed following the figure or combined and placed after the main text of the manuscript.

Response: The *PLOS ONE* submission guidelines instructed that the figure legends should be provided separately from the figures. As per the guidelines, figures should not be included in the main manuscript file. Accordingly, we have provided separate figures in the formats specified by the guidelines.

9. Virus loads in BALFs and lungs were determined on day 3 after IFV infection and neutralizing antibody and IgA levels were determined on day 14. Have you tested the virus load in BALFs and lungs on day 3 as well?

Response: The comment was slightly ambiguous. If your query is regarding virus detection on day 14 of infection, no virus was detected on day 14 in the all groups. The virus was not detected in both BALF and lung samples by day 7 after virus inoculation.

10. In Figure 3, a and b seems showing same magnification based on the scale bar, however the images seem to be taken under obviously different magnification scale, this should be addressed and corrected, if any applicable. Additionally, the indications of different fluorescence colors presenting in Figure 3c should be added in the figure legend.

Response: The images (a) and (b) in Fig 3 are of the same magnification. However, n-LAB in (a) appears to be larger because of the agglomeration; thus, the magnification may be perceived differently. Therefore, in the Results section, we have added that Fig.3a shows a larger bacterial image than Fig.3b owing to bacterial aggregation [L190-191]. Different fluorescent color indications have been added to the figure legend.

11. Line 181-184. This part of result was written poorly. The rationale and hypothesis of this specific experiments set should be addressed and a conclusion from the observations should be made or at least discussed.

Response: We agree that we needed to clarify the rationale of the experiment. We have made corrections and additions in the Introduction and Discussion sections for clarity [L77-78, L286-287].

12. Influenza infected mice treated with d-LAB lost weight at a slower rate and recovered faster compared to those treated with n-LAB, was the difference statistically significant? The signifiers should be added to the graphs if there is any.

Response: No significant difference was found. However, because it was an interesting pattern, we have added the number of days required to return to pre-infection weight for the Control, n-LAB, and d-LAB groups [L221-224].

13. Figure 6a, significance bar misplaced. Please correct this.

Response: Thanks for bringing this to our notice. We have rectified it.

14. Line 207, The d-LAB group had a significantly lower viral load than n-LAB group in the BALFs but no differences were seen in the lung virus yields, can you explain this?

Response: There was a substantial variation between individual mice of the d-LAB group in terms of the measured virus yields. We presumed that this variation is the cause for the difference observed.

15. A control group of influenza infected mice treated with LAB culture preparation without powderization should be added, in comparison to different types of powdered LAB preparations.

Response: Thank you for your comments. We agree and consider it to be important as well. However, because this study was conducted with limited resources, we would like to compare and verify the results with LAB culture preparation without powderization and with LAB powderized using different methods in our ongoing research. We added an explanation about this in the Discussion [L296-297].

16. Are there any other aspects other than the formation of aggregates that could possibly affect the beneficial effects of LAB? (eg, bacteria morphology changes during and after powderization, biofilm formation, et al.)

Response: Thank you for your targeted question. We believe that heat sterilization temperature may be an important factor. We are currently preparing heat-killed lactic acid bacteria with different sterilization temperatures and studying the immune response in vitro and in vivo. We are confirming the difference depending on the sterilization temperature and will report the results at the earliest.

We thank you for giving us the opportunity to strengthen our manuscript with your valuable comments and queries.
